# An Incidental Discovery of the Tibiocalcaneus Internus Muscle of Testut: An Exceedingly Rare Variation of the Soleus Muscle

**DOI:** 10.7759/cureus.84925

**Published:** 2025-05-27

**Authors:** Mary Walker, Hosne Ara, Adegbenro O Fakoya

**Affiliations:** 1 Cellular Biology and Anatomy, Louisiana State University Health Sciences Center, Shreveport, USA

**Keywords:** accessory soleus, anomalous muscle, anterior tarsal tunnel syndrome, cadaver case report, posterior compartment of the lower limb, tibiocalcaneus internus muscle

## Abstract

This report describes an incredibly rare variant of the soleus, the tibiocalcaneus internus muscle of Testut, observed during routine cadaveric dissection. In our cadaveric dissection, we observed this tibiocalcaneus internus muscle of Testut as an accessory muscle in the posterior compartment of the leg, which originates at the soleal line of the tibia and travels deep to the flexor retinaculum, ending in a tendinous insertion on the medial surface of the calcaneus, medial to the tendon of Achilles. Due to the few existing reports of this anomalous finding, any occurrence warrants reporting to expand the knowledge in clinical practice. Although it remains asymptomatic during the lifetime, this variant muscle may have clinical relevance, particularly in diagnostic imaging and in cases of unexplained medial ankle pain. By understanding this rare anatomical variation, clinicians, surgeons, and radiologists could avoid misdiagnosis and unnecessary interventions.

## Introduction

The muscles of the posterior compartment of the lower limb play a crucial role in facilitating plantar flexion of the foot, contributing significantly to essential motor functions such as walking, running, and stair climbing. Anatomical variations in this region can have significant clinical implications, potentially leading to vascular or neural impingement and associated pain or dysfunction [1,2].

The primary muscles within the superficial compartment of the posterior leg include the gastrocnemius, plantaris, and soleus muscles. Typically, the soleus originates from the fibula and medial border of the tibia and inserts into the calcaneus via the calcaneal (Achilles) tendon [3]. Although variations of the soleus muscle are relatively rare, multiple anatomical studies have documented its anomalies, including partial or complete absence and duplication of its tibial or fibular head. In cases of duplication, the additional muscle component often presents as an accessory soleus, which lies deep to the primary soleus muscle [1,4,5].

The accessory soleus muscle most commonly originates from the soleal line of the tibia. However, alternative origins from the deep surface of the soleus muscle or the proximal third of the fibula have also been reported [6-8].

In 2011, Hatzantonis et al. described the trajectory of the accessory soleus, noting that it typically runs anterior or anteromedial to the calcaneal tendon and exhibits three distinct types of distal insertions. There are three types based on the insertion pattern: Type A - onto the calcaneus medial to the Achilles tendon via a separate tendon; Type B - directly into the Achilles tendon; and Type C - onto the calcaneus medial to the Achilles tendon via a bifurcated tendinous or fleshy connection [8].

Additionally, an earlier study by Jean Léo Testut (1884) described a rare soleus muscle variant, morphologically similar to the Type C accessory soleus. This muscle, the tibiocalcaneus internus muscle of Testut, forms a direct connection between the tibia and calcaneus [9]. Since its initial description, only a few cases have been documented in the anatomical literature.

In this cadaveric case study, we report the presence of this exceptionally rare variant, detailing its morphology and clinical implications. Recognizing such anatomical variations is critical for improving the accuracy of clinical diagnoses, optimizing surgical approaches, and preventing misinterpretation of imaging findings.

## Case presentation

This anatomical variation was identified during a routine cadaveric dissection of the left lower limb of a 75-year-old male cadaver at Louisiana State University Health Sciences Center, Shreveport, USA. Following standard dissection protocols, the skin and fascia were systematically resected. The great saphenous vein was preserved, whereas all other superficial arteries, veins, and nerves were excised to enhance visualization of the underlying musculature.

**Figure 1:**
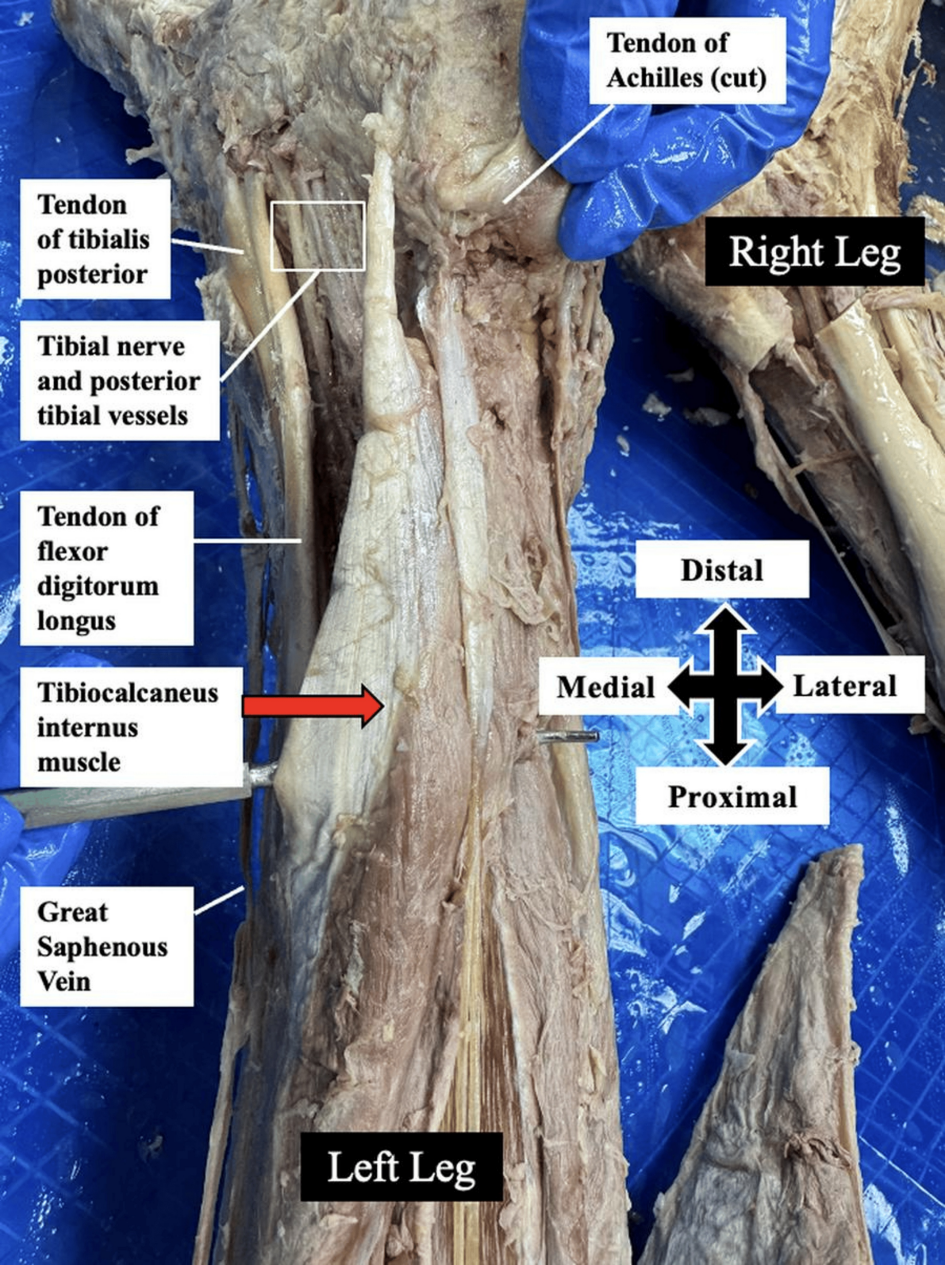
Posteromedial view of the left leg This figure shows the gastrocnemius and soleus muscles transected at the distal Achilles tendon and dissected away from the deeper structures, revealing the tibiocalcaneus internus muscle (positioned over the probe and indicated by a red arrow).

**Figure 2:**
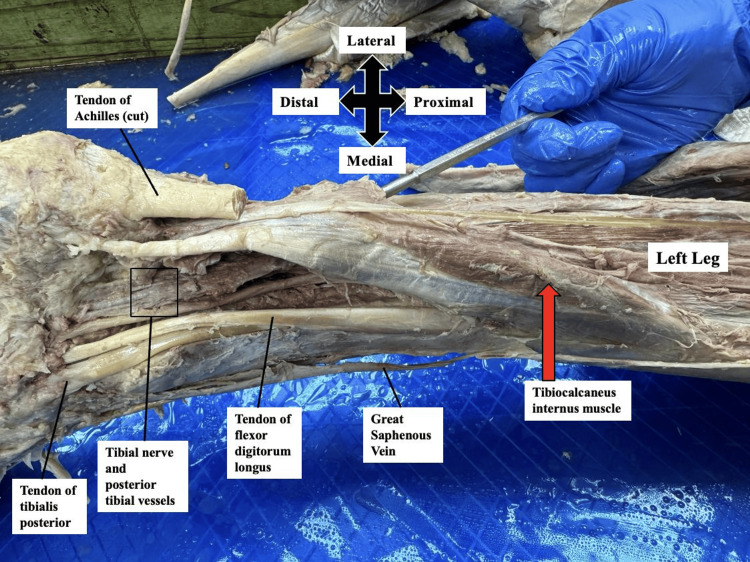
Posteromedial view of the left leg This figure shows the posteromedial view of the tibiocalcaneus internus muscle (red arrow), running deep to the gastrocnemius and soleus (removed), with insertion medial to the Achilles tendon.

After dissecting the superficial structures, the gastrocnemius and soleus muscles were transected at their distal attachments to the Achilles tendon and carefully reflected to expose the deep compartment of the posterior leg. This dissection revealed the presence of an anomalous tibiocalcaneus internus muscle (Figures 1-2).

The tibiocalcaneus internus muscle was observed to originate along the medial border of the mid-shaft of the tibia. It coursed inferiorly, running deep to the gastrocnemius and soleus muscles, and parallel to the tibialis posterior muscle. Distally, the muscle passed beneath the flexor retinaculum, which was dissected out, before terminating in a tendinous insertion on the medial surface of the calcaneus. It crossed the posterior tibial vessels and the tibial nerve along its course to insertion, as shown in Figures 1-2. This neurovascular bundle supplies the muscle and all the posterior leg compartment muscles. No similar muscular anomaly was identified in the contralateral limb.

## Discussion

The tibiocalcaneus internus muscle of Testut represents the rarest documented variant of the soleus muscle, with only a few cases reported in the anatomical literature. This variant closely resembles the Type C accessory soleus muscle; however, its distinct anatomical course differentiates it from other accessory soleus variations. To fully understand the identification of this rare anomaly, it is necessary to review the classification of accessory soleus muscles.

Accessory soleus muscles are categorized into three subtypes - Type A, Type B, and Type C - based on their mode of insertion. All three types may originate from the proximal third of the fibula, the soleal line of the tibia, or the anterior surface of the soleus muscle. Their distal attachments distinguish them. Type A inserts onto the calcaneus medial to the Achilles tendon via a separate tendon. Type B inserts directly into the Achilles tendon. Type C attaches to the calcaneus medial to the Achilles tendon via a bifurcated fleshy or tendinous connection. In our cadaveric case, the anomalous muscle was inserted onto the calcaneus medial to the Achilles tendon, initially suggesting a Type A or Type C accessory soleus muscle (with bifurcation). However, further dissection revealed that the muscle traveled deep to the flexor retinaculum, distinguishing it from typical accessory soleus muscles, which course superficially to the retinaculum. This deep trajectory confirms its classification as the rare tibiocalcaneus internus muscle of Testut [10].

Due to the inherent limitations of cadaveric studies, the functional role of the tibiocalcaneus internus muscle remains speculative. However, based on its anatomical positioning, it is hypothesized to assist the tibialis posterior muscle in stabilizing the medial longitudinal arch of the foot. A 1994 case report by Sammarco and Conti documented a patient with a tibiocalcaneus internus muscle of Testut who developed tarsal tunnel syndrome, a neuropathic condition caused by compression of the posterior tibial neurovascular bundle. This syndrome manifests as exercise-induced pain, with severity ranging from mild discomfort to significant functional impairment [11].

Radiological assessment is crucial for accurately diagnosing accessory soleus muscles and their variants. Magnetic resonance imaging (MRI), computed tomography (CT), and ultrasound are commonly employed for evaluation. However, unfamiliarity with this rare anatomical variant may result in misdiagnosis as a soft-tissue mass or other pathology. Among available imaging modalities, MRI has been reported as the most reliable, as it allows differentiation between accessory muscles and soft-tissue neoplasms. Therefore, MRI should be considered in patients with unexplained posterior lower limb masses [12].

In cases where tarsal tunnel syndrome arises due to the tibiocalcaneus internus muscle of Testut, surgical resection has been reported as an effective intervention. Excision of the anomalous muscle can relieve compression within the flexor retinaculum, alleviating pain and improving patient outcomes [11].

Given the potential for neuropathic pain syndromes related to this rare muscle variant, clinicians should maintain a high index of suspicion when evaluating persistent tibial nerve-related symptoms. By documenting this case, we aim to increase awareness of this rare anatomical anomaly, enhancing the ability of radiologists, surgeons, and healthcare providers to diagnose and manage similar cases accurately.

## Conclusions

Identifying the tibiocalcaneus internus muscle of Testut in this cadaveric dissection underscores the importance of recognizing rare anatomical variants within the posterior compartment of the leg. Although often asymptomatic, such variants may have significant clinical implications, particularly in the context of diagnostic imaging or in patients presenting with unexplained posterior ankle or medial foot pain. This case reinforces the need for clinicians, radiologists, and surgeons to be aware of the tibiocalcaneus internus muscle to avoid potential misdiagnosis, such as mistaking it for a soft tissue mass, and to manage cases of tarsal tunnel syndrome appropriately. By contributing to the limited literature on this rare anomaly, our report supports the value of cadaveric studies in enhancing anatomical knowledge and improving patient outcomes through better-informed clinical decision-making.
